# Determination of the Molecular Weight of Low-Molecular-Weight Heparins by Using High-Pressure Size Exclusion Chromatography on Line with a Triple Detector Array and Conventional Methods

**DOI:** 10.3390/molecules20035085

**Published:** 2015-03-19

**Authors:** Antonella Bisio, Alessandra Mantegazza, Davide Vecchietti, Donata Bensi, Alessia Coppa, Giangiacomo Torri, Sabrina Bertini

**Affiliations:** 1 Istituto di Ricerche Chimiche e Biochimiche G. Ronzoni, via G. Colombo 81, Milan 20133, Italy; E-Mails: ale.ciuni@gmail.com (A.M.); davide.vecchietti@unimi.it (D.V.); torri@ronzoni.it (G.T.); bertini@ronzoni.it (S.B.); 2 Laboratori Derivati Organici, via M. Barozzi 4, Milan 20122, Italy; E-Mails: donata.bensi@ldospa.it (D.B.); alessia.coppa@ldospa.it (A.C.)

**Keywords:** low molecular weight heparins, size exclusion chromatography, laser light scattering

## Abstract

The evaluation of weight average molecular weight (Mw) and molecular weight distribution represents one of the most controversial aspects concerning the characterization of low molecular weight heparins (LMWHs). As the most commonly used method for the measurement of such parameters is high performance size exclusion chromatography (HP-SEC), the soundness of results mainly depends on the appropriate calibration of the chromatographic columns used. With the aim of meeting the requirement of proper Mw standards for LMWHs, in the present work the determination of molecular weight parameters (Mw and Mn) by HP-SEC combined with a triple detector array (TDA) was performed. The HP-SEC/TDA technique permits the evaluation of polymeric samples by exploiting the combined and simultaneous action of three on-line detectors: light scattering detectors (LALLS/RALLS); refractometer and viscometer. Three commercial LMWH samples, enoxaparin, tinzaparin and dalteparin, a γ-ray depolymerized heparin (γ-Hep) and its chromatographic fractions, and a synthetic pentasaccharide were analysed by HP-SEC/TDA. The same samples were analysed also with a conventional HP-SEC method employing refractive index (RI) and UV detectors and two different chromatographic column set, silica gel and polymeric gel columns. In both chromatographic systems, two different calibration curves were built up by using (i) γ-Hep chromatographic fractions and the corresponding Mw parameters obtained via HP-SEC/TDA; (ii) the whole γ-Hep preparation with broad Mw dispersion and the corresponding cumulative distribution function calculated via HP-SEC/TDA. In addition, also a chromatographic column calibration according to European Pharmacopoeia indication was built up. By comparing all the obtained results, some important differences among Mw and size distribution values of the three LMWHs were found with the five different calibration methods and with HP-SEC/TDA method. In particular, the detection of the lower molecular weight components turned out to be the most critical aspect. Whereas HP-SEC/TDA may underestimate species under 2 KDa when present in low concentration, other methods appeared to emphasize their content.

## 1. Introduction

Heparin is a complex and highly negatively charged sulfated polysaccharide belonging to the glycosaminoglycan family, well known for its medical importance as anticoagulant and antithrombotic drugs. Heparin linear chains are made up of repeating disaccharide units consisting of N-sulfated or N-acetylated α–d–glucosamine α1→4 linked to hexuronic (β–d–glucuronic or α–l–iduronic) acid, both monosaccharides bearing O-sulfates at varying positions. Pharmaceutical grade unfractionated heparin (UFH) preparations are polydisperse mixtures of molecules ranging in molecular weight (Mw) from less than 6000 to over 60,000 Da [1,2]. Such a high degree of polydispersity together with the overall sulfation pattern confers to heparin a remarkable level of structural heterogeneity. To minimize some unwanted side effects of UFH, such as bleeding and thrombocytopenia, and improve some pharmacological properties, as bioavailability and duration of therapeutic action, low molecular weight heparins (LMWHs) were developed [3].

LMWHs are derived from UFH by controlled chemical, physical, or enzymatic depolymerisation to yield fragments that are approximately one third of the original chains, with Mw ranging from 1000 to 10,000 Da [4,5]. Depending on the manufacturing process, LMWHs mainly differ in degree of depolymerisation and in chemical structure of end-units, as well as in therapeutic and pharmacological properties. Since chain length is one of the parameters highly affecting LMWH biological activity, an accurate determination of molecular weight distribution is particularly important.

The evaluation of weight average molecular weight (Mw) and molecular weight distribution is one of the most controversial aspects concerning the characterization of LMWHs. As the most commonly used method for the measurement of such parameters is high performance size exclusion chromatography (HP-SEC), the soundness of results mainly depends on the appropriate calibration of chromatographic columns.

A series of reference standards have been explored including heparin fractions with narrow molecular weight distribution [6,7], a partially depolymerized heparin sample with a broad polydispersity [8], and pullulan fractions [9]. A chromatographic method for determining LMWH Mw distribution based on multi-angle laser light scattering (MALLS) technology in conjunction with HP-SEC was developed [10,11]. This method overcame the critical dependence on adequate calibration products and improved the reliability of response with respect to the one previously proposed by Komatsu *et al.* that exploited low angle laser light scattering (LALLS) as detector [12]. Recently HP-SEC on-line with a multiple detector array (TDA) was successfully employed to determine molecular weight of UFH and dermatansulfates [1]. Also this approach, which exploits the combined and simultaneous action of a dual angle laser light scattering, refractometer and viscometer, does not require any chromatographic column calibration.

In the present work, the comparison of molecular weight distribution of samples of three commercial LMWHs, enoxaparin, tinzaparin and dalteparin and of a synthetic pentasaccharide, fondaparinux, measured via HP-SEC/TDA and conventional HP-SEC/Refractive Index (RI) method was performed. For this latter method, column calibration was achieved by using (i) a sample of heparin partially depolymerised by γ-rays (γ-Hep) [13]; (ii) several fractions obtained from γ-Hep characterised by narrow Mw distribution; and iii) the European Pharmacopoeia (EP) LMWH reference standard. Both broad Mw sized γ-Hep and its narrow Mw sized fractions were characterized by HP-SEC/TDA. Moreover, within the conventional HP-SEC/RI method, two different chromatographic conditions were applied, *i.e.*, polymeric gel columns according to HP-SEC/TDA method, and silica gel columns according to EP.

## 2. Results and Discussion

### 2.1. Characterization of HP-SEC Calibrants

**Figure 1:**
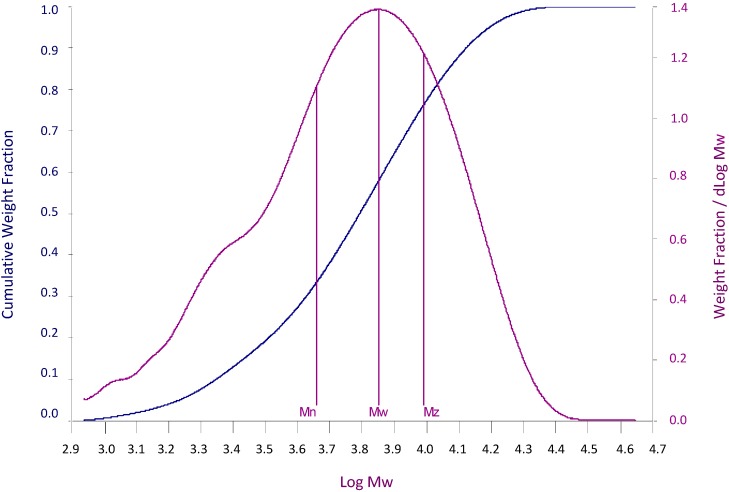
Curve of molecular weight distribution of partially depolymerized heparin (γ-hep) together with the cumulative distribution function, generated by HP-SEC/TDA method.

A partially depolymerised heparin mixture, γ-Hep [13], obtained by controlled y-ray irradiation treatment of a pig mucosal heparin, was characterised for its molecular weight distribution, Mn (number average molecular weight), Mw (weight-average molecular weight) and Pd (polydispersity calculated as Mw/Mn ratio). In particular Mw is 7100 Da, Mn 4800 Da and the Pd 1.61. On consideration of its wide polydispersity, γ-Hep was regarded as a broad calibrant and its cumulative distribution function was calculated, by expressing the relative molecular mass, log(M_r_), *vs.* the cumulative percent of sample above that Mw. As reported in Table 1, log(M_r_) values ranged from 2.991 to 4.258. This table was used to generate a direct calibration curve of Mr *vs.* retention volume (Vr) by appropriated gel permeation chromatography software. In Figure 1, the molecular weight distribution curve together with the cumulative distribution function is reported.

**Table 1:** Broad calibration table of a partially depolymerised heparin mixture, obtained by controlled γ-ray irradiation treatment of a pig mucosal heparin (γ-Hep).

Point	Cum.%	log (M_r_)	M_r_
1	2.1	4.258	18,100
2	3.1	4.228	16,900
3	5.2	4.182	15,200
4	6.9	4.155	14,300
5	9.1	4.124	13,300
6	12.2	4.090	12,300
7	17.6	4.037	10,900
8	33.7	3.914	8200
9	41.8	3.857	7200
10	51.0	3.792	6200
11	61.0	3.716	5200
12	71.0	3.623	4200
13	80.4	3.505	3200
14	88.5	3.342	2200
15	96.8	3.079	1200
16	98.1	2.991	980

The γ-Hep sample was then fractionated by size exclusion chromatography into 29 fractions. All fractions were characterised by HP-SEC/TDA and 18 of them were selected and, on consideration of their low degree of polydispersity, were regarded as narrow standards. The molecular weight values (Mn, Mw, Mp and Pd) of each fraction, obtained with OmniSEC software data analysis, are reported in Table 2. In addition to selected fractions, a heparin disaccharide, 2-O-sulfated-α-l-iduronic acid 1→4 linked to 6-O-sulfated anhydromannitol residue (molecular mass 566 Da) was used for calibration purpose.

**Table 2:** Characterization by HP-SEC/TDA method of eighteen selected narrow cut fractions obtained from γ-hep by size exclusion chromatography. Mp value of the heparin derived disaccharide is also reported.

Fraction	Vr ^#^	Mp	Mn	Mw	Pd
1	11.70	18,776	15,323	17,615	1.15
2	11.86	14,646	13,778	15,256	1.10
3	11.93	13,046	12,173	13,383	1.10
4	12.14	9815	9444	10,262	1.09
5	12.17	9231	9183	9676	1.05
6	12.18	8390	8332	8714	1.05
7	12.31	6883	6862	7151	1.04
8	12.35	6286	6023	6482	1.07
9	12.48	5410	5044	5666	1.12
10	12.49	4663	4500	4796	1.07
11	12.64	3871	3686	3881	1.05
12	12.75	3554	3476	3599	1.04
13	12.93	2808	2713	2824	1.04
14	13.05	2653	2501	2631	1.05
15	13.19	2192	2059	2198	1.07
16	13.29	2022	2006	2098	1.05
17	13.47	1830	1746	1827	1.05
18	13.82	1198	1369	1479	1.08
19		566			

^#^ retention volume (mL).

For all calculations, a differential refractive index increment (dn/dc) value of 0.120 was used for converting RI voltages to solute concentration at each data slice across a chromatographic peak. It was calculated as previously described [1]. Full recovery was detected for all the depolymerised heparin fractions by RI signal, which is proportional to the sample concentration and to the RI increment (dn/dc), indicating that no adsorption of material to the columns occurred and no low molecular weight components were eluted together with the salt peak.

### 2.2. Calibration of the HP-SEC Systems

**Figure 2:**
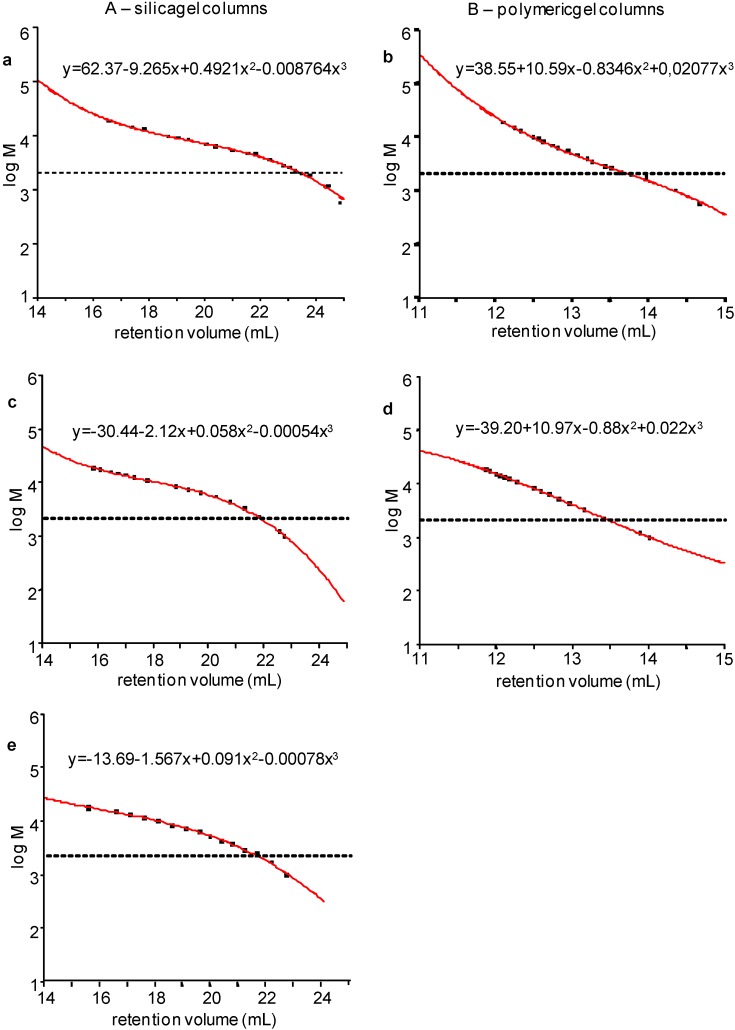
Typical calibration curves of (A) silica gel columns with narrow fractions (**a**), broad γ-hep (**c**) and EP method (**e**), and (B) polymeric gel columns with narrow fractions (**b**) and broad γ-hep (**d**).

Calibration curves with both the broad dispersed γ-Hep and the series of its narrow dispersed fractions, based on data of Table 1 and Table 2 respectively, were built up on conventional HP-SEC/RI-UV system by applying two different chromatographic conditions: (i) according to HP-SEC/TDA procedure, by employing polymeric gel columns and 0.1 M NaNO_3_ as eluent; (ii) according to the EP indications [14], by using silica gel columns and 0.2 M Na_2_SO_4_ as eluent. In addition, a calibration curve with the EP Reference standard, according to EP indications [14], was also implemented. The five calibration curves obtained are presented in Figure 2, together with the equation of the fitted curves.

The first relevant difference between silica gel and polymeric columns is due to the different retention volumes: experimental points appear distributed over 8 mL for the former and over 2–3 mL for the latter.

### 2.3. Measurement of Molecular Weight Parameters of LMWHs

#### 2.3.1. HP-SEC/TDA

**Figure 3:**
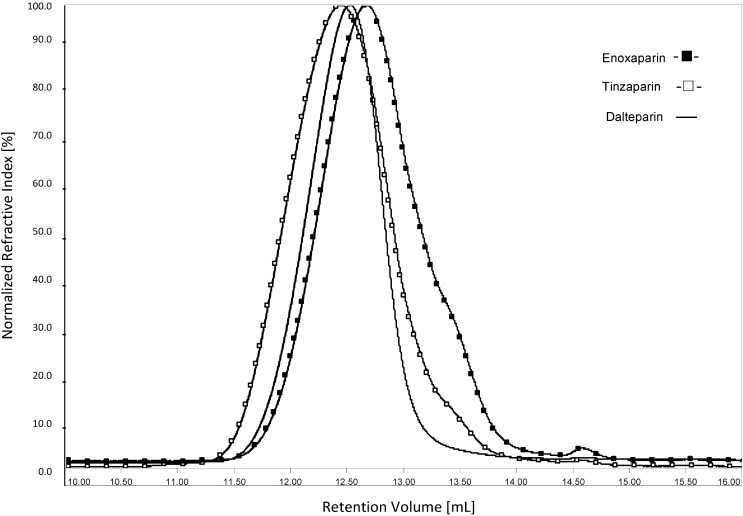
HP-SEC/TDA chromatograms (RI response *vs.* retention volume) of enoxaparin, tinzaparin and dalteparin.

The RI elution profiles of enoxaparin, tinzaparin, and dalteparin obtained through HP-SEC/TDA are displayed in Figure 3. In agreement with the observed compositional differences of the three LMWHs regarding the relative content of the different oligomeric components [5], their elution profiles differ both in retention time and in broadness. The resulting molecular weight parameters (Mw, Mn, and Pd), obtained with OmniSEC software data analysis, were determined by subjecting each sample to five independent analyses, each with a double run, over a time span of 18 months. The average results, together with standard deviation of Mw and Mn values are presented in Table 3. The error of determinations was below 3.5%. The error of Mw determination, for four runs within the same day, was below 1.2%.

**Table 3:** Characterization of three LMWHs by HP-SEC/TDA method.

LMWH	Mn	Mw	Pd
Enoxaparin	4036 ±123	5426 ± 101	1.34
Tinzaparin	5920 ± 207	8270 ± 235	1.40
Dalteparin	5668 ± 96	6910 ± 131	1.22

Since one of the purpose of the present work is the comparison of the above data with the results obtained from silica column chromatography with 0.2 M Na_2_SO_4_ as eluent (see the Experimental section), a check of the possible influence of the different ionic strength (0.1 M *vs.* 0.2 M of NaNO_3_) on chromatographic resolution of sample was performed. The three LMWHs were subjected to HP-SEC/TDA analysis on polymeric gel columns using 0.1 M and 0.2 M NaNO_3_, in both cases very similar Mw and Pd values were obtained (data not shown).

#### 2.3.2. HP-SEC/RI and HP-SEC/RI-UV

The three LMWHs were also analysed by conventional HP-SEC equipped with RI detector using both polymeric and silica gel columns and 0.1 M NaNO_3_ as eluent. Mw, Mn and Pd were calculated by applying the calibrations curves built up with both narrow fractions and broad calibrant on the corresponding set of chromatographic columns. In addition, enoxaparin, tinzaparin, and dalteparin were also analysed according to the EP indications [14] as previously described and the corresponding molecular weight parameters (Mw and Pd) were calculated by applying the calibration curve built up with the EP reference standard. All the results, calculated as a mean of a double run, are shown in Table 4, in comparison with the corresponding data obtained with HP-SEC/TDA method. In all cases, the percentage of chains with Mw lower than 2 KDa, or than 3 KDa for dlteparin, between 2 and 8 KDa, and higher than 8 KDa are given.

**Table 4:** Comparison of the molecular weight profiles of enoxaparin, tinzaparin, dalteparin and fondaparinux obtained by HP-SEC/TDA method, and conventional HP-SEC/RI or HP-SEC/RI-UV by applying five different calibration methods: with narrow fractions or broad calibrant on both polymeric and silica gel columns, and with the EP standard on silica gel columns. Mw values were rounded off to the nearest hundred for LMWHs and to the nearest ten for fondaparinux. (nd: not determined).

LMWH	Detector	Calibration	Stationary Phase	Mw	Pd	% Mw			
						<2000	<3000	2000–8000	>8000
Enoxaparin	TDA	-	polymer	5400	1.34	7	nd	76	17
	RI	narrow fr.	polymer	4900	1.39	12	nd	76	13
			silica	5000	1.35	8	nd	82	10
		broad cal.	polymer	5300	1.45	11	nd	72	17
			silica	4800	1.60	19	nd	65	16
	RI-UV	EP std	silica	4600	1.59	22	nd	65	14
Tinzaparin	TDA	-	polymer	8300	1.40	2	nd	56	42
	RI	narrow fr.	polymer	7300	1.48	5	nd	62	34
			silica	7600	1.36	3	nd	68	30
		broad cal.	polymer	7900	1.45	4	nd	56	40
			silica	7900	1.57	6	nd	52	42
	RI-UV	EP std	silica	7400	1.59	8	nd	53	39
Dalteparin	TDA	-	polymer	6900	1.22	nd	5	68	30
	RI	narrow fr.	polymer	6400	1.26	nd	9	75	23
			silica	6600	1.14	nd	2	81	18
		broad cal.	polymer	7200	1.23	nd	4	66	33
			silica	6900	1.28	nd	5	66	32
	RI-UV	EP std	silica	6600	1.32	nd	nd	69	29
Fondaparinux	TDA	-	polymer	1890	1.01	97			
	RI	narrow fr.	polymer	2030	1.04	46			
			silica	2080	1.01	28			
		broad cal.	polymer	1920	1.08	55			
			silica	1410	1.02	100			
	RI-UV	EP std	silica	1420	1.02	100			

For each LMWH, the Mw values measured with all the different calibration methods varied within a narrow range, the maximum variation being around 800 Da for enoxaparin, 1000 Da for tinzaparin and 800 Da for dalteparin. More consistent discrepancies were observed for Pd values.

In case of employment of broad calibrants (γ-Hep or EP standard) with silica columns, all the three LMWHs exhibited the highest Pd values. Such a result turned out to be in agreement with the declared high resolution capacity of silica gel columns with respect to polymeric columns. Nevertheless, by employing narrow calibrants Pd values obtained with silica columns were found lower than those obtained with polymer columns, for each LMWHs.

Very low molecular weight species are significantly represented only in enoxaparin, whereas in tinzaparin and dalteparin they are less important or negligible, respectively. Different amount of species < 2 KDa were determined in enoxaparin depending on the method applied (Table 4). The lowest value found by HP-SEC/TDA (7) probably reflected the lower sensitivity of light scattering detector toward very low molecular weight molecules. The reliability of the other values detected was considered in relation to their corresponding calibration curves (Figure 2). As concerns broad calibration and EP method on silica gel columns (Figure 2c,e, respectively) a very critical part of the curve is over 22 mL of retention volume, where the deviation from linearity clearly appears. Most probably the high values detected in these cases (19 and 22, respectively) derived from an overestimation of very low molecular weight oligosaccharides. In the case of calibration with narrow standard on silica gel columns, such as narrow standards and broad standard on polymeric gel columns, the curves maintained a good linearity in the region of low molecular weight species and the percentage values of chains under 2 KDa were lower, *i.e.*, 8, 12 and 11, respectively.

To verify the hypothesis of the overestimation, enoxaparin was fractionated on Biogel P10 as previously described [5] and fractions under 2 KDa, *i.e.*, tetramers and hexamers, were collected, desalted and weighted after freeze-drying. Their total percentage with respect to the whole sample turned out to be around 10%, thus confirming the overestimation of the previously detected 20% (data not shown). Larger oligosaccharide components were not considered since LC/MS analysis of both the whole enoxaparin and the octasaccharide peak from Biogel P10 did not detect heptamers or octamers under 2 KDa in significant amount, as reported by Alekseeva *et al.* [15], and in Figure S1 (Supplementary Material), respectively.

Accordingly, 2 KDa represents a very critical limit for the quantification of underneath oligomeric species. Very slight variations of the calibration curve slope, even if non influential on Mw determination, can result in consistent differences of the percentage determination of chains with Mw values lower than 2000 Da.

According to EP monograph, the three commercial LMWHs enoxaparin, tinzaparin and dalteparin, are identified by the mass-average Mw ranges and molecular weight distribution values reported in Table 5 [14]. By comparing our results with EP requirements, only a few series of the results obtained with the different methods, reported in Table 4, fully satisfy the requirements of the EP. This was the case of HP-SEC/RI method with polymeric gel columns calibrated with narrow fractions: both mass-average Mw and Mw distribution values of the three LMWHs turned out to be included in the required ranges. In all other cases at least one of the measured parameters failed to meet the reported ranges. However the enoxaparin data obtained using RI and RI-UV methods with the γ-Hep and EP standard respectively were quite close to EP requirement. As regards EP method, it is worthwhile to highlight that its implementation was particularly demanding. Calibration of the GPC system with EDQM broad standard turned out to be a critical aspect. Its chromatographic profile and peak resolution mostly depend on the quality of silica gel columns, whose fragile stationary phase can vary both over time and from batch to batch, thus affecting their resolving power. Accordingly, the alignment of UV and RI chromatographic profiles of the standard sometimes did not allow an unequivocal assignment of the degrees of polymerization to the oligosaccharide peaks, so that the addition of pure di- or oligosaccharide species became necessary for a precise calibration.

**Table 5:** Mass-average Mw range and molecular weight distribution of the three commercial LMWH as reported in EP monograph [14].

LMWH	Mass-Average Mw Range	Molecular Weight Distribution			
		<2000 Da	<3000 Da	2000–8000 Da	>8000 Da
Enoxaparin	3800–5000	12%–20%	-	68%–82%	-
Tinzaparin	5500–7500	≤10%	-	60%–72%	22%–36%
Dalteparin	5600–6400	-	≤13%	-	15%–25%

#### 2.3.3. Measurement of Molecular Weight Parameters of a Synthetic Pentasaccharide

The molecular weight parameters (Mw and Pd) of the synthetic pentasaccharide fondaparinux (Mw = 1728 Da) were calculated by applying all the calibration curves above described. Results presented in Table 4 indicate that the TDA method can measure LMW species when present in adequate amounts. For conventional SEC, besides on the soundness of column calibration, a reliable quantitative evaluation of smaller oligomeric species depends also on the minimization of the peak broadening effect, especially in the region of very low molecular weights [16]. To minimize such a phenomenon, which is observable also for mono-dispersed species (e.g., fondaparinux), highly efficient chromatographic columns are required.

From the overall results, it appears that Mw and Pd values obtained are strictly dependent on the measurement method applied. In particular, the calibration of polymeric gel columns with a selected series of well characterized heparin derived narrow standards guaranteed the correct evaluation of molecular weight parameters of LMWHs, according to EP requirement. In addition, the stable stationary phase of polymeric columns permitted high reproducibility of analyses even over a long time span. Nevertheless the preparation of narrow standards is an involved and time consuming work. In principle, the application of the cumulative distribution function of a broad heparin standard represents a simpler and efficient tool for calibration, providing the size distribution of the standard is sufficiently wide and adequate for the mass range of the analysis samples. Actually, broad calibration especially on silica gel columns turned out to give positive results. Unfortunately, silica gel columns, together with a high resolution mainly of very low Mw species, exhibited a short lifetime due to a slight but continuous leakage of stationary phase, which can induce a noisy baseline, depending on the detector employed. HP-SEC/TDA method, besides to appear reproducible over a long period, have been successfully employed for characterizing both narrow and broad heparin derived standards. HP-SEC/TDA gave for dalteparin Mw very close to values obtained with all other methods, whereas for enoxaparin and tinzaparin it gave the highest Mw values. As the lower molecular weight components, *i.e.*, tetramers and hexamers, are particularly represented in enoxaparin and tinzaparin [5], such a discrepancy is partly due to an underestimation of those heparin chains by TDA, and partly by an overestimation of the same species by other methods, such as EP method. Actually, the lower sensitivity of TDA appeared only when very low molecular weight species were present in low concentration. Indeed HP-SEC/TDA turned out to be the best procedure for detecting the Mw and Pd values of a synthetic pentasaccharide.

## 3. Materials and Methods

### 3.1. Materials

LMWH preparations used in this study were enoxaparin, supplied by Sanofi-Aventis Pharma (Milan, Italy) as injectable Clexane; tinzaparin, supplied by LEO-Pharma (Ballerup, Denmark) as a powder; dalteparin, from Pharmacia AB (Uppsala, Sweden) as injectable Fragmin. The synthetic pentasaccharide Arixtra (fondaparinux) was from GlaxoSmithKline (Verona, Italy). Heparin low-molecular-mass for calibration *CRS* batch 1, (Mn = 3700 Da) was purchased from European Pharmacopoeia (EP) LMWH standard.

### 3.2. Fractionation of Depolymerized Heparin

250 g of pig mucosal heparin partially depolymerised by γ-rays [13], dissolved in 0.3 M NaCl, 0.02% NaN_3_ (*w*/*v*) at a final concentration of 150 g/L were loaded onto gel permeation column (Sephadex G-50, GE Healthcare, Uppsala, Sweden; 35 × 100 cm) and eluted with 78 L of the same solution, by collecting 1.62 mL fractions. The eluate, based on the absorbance at 210 nm profile, was divided into 29 fractions that were desalted and freeze-dried.

### 3.3. HP-SEC/TDA Analysis

The HPLC equipment consisted of a Viscotek system (Malvern Instrument Ltd, Malvern, UK) equipped with a VE1121 Solvent Delivery pump, and a Gastorr 150 metal free two channel on-line degassing device. The detector system was a Viscotek mod. 302 Triple Detector Array (TDA), which is composed as previously described [1] except for the additional presence of Low Angle Laser Light Scattering (LALLS) detector, with the following technical specifications: measuring Angle 90° and 7°, constant optical power output laser diode Light source, wavelength 670 nm; detectors Photodiode/Amplifier and Cell Volume 10 μL.

Two TSK gel columns in series, G3000 PWXL (7.8 × 300 mm) and G2500 PWXL (7.8 × 300 mm) (Tosoh Corp., Tokyo, Japan), were used. Columns, injector and detectors were maintained at 40 °C. An aqueous solution of 0.1 M NaNO_3_ pre-filtered on 0.22 µm filter (Merck Millipore, Darmstadt, Germany) was used as mobile phase at a flow rate of 0.6 mL/min. The system was calibrated with the polyethylene oxide (PEO) narrow standard of known Mw, polydispersity and intrinsic viscosity. Analysis of data was performed with OmniSEC software, version 4.0 (Viscotek).

For all calculations, a differential refractive index increment (dn/dc) value of 0.120 was used for converting RI voltages to solute concentration at each data slice across a chromatographic peak, as previously described [1].

### 3.4. HP-SEC/RI-UV Analysis

Conventional HP-SEC analyses were performed with a Waters 515 pump (Water Inc., Milford, MA, USA), equipped with an auto-injector (Waters717plus), ultraviolet (UV) detector (Waters 2487) and differential refractometer (RI) detector (Waters 2410). Both polymeric gel based columns and silica based columns were used. In the first case, TSK G3000 PWXL (7.8 × 300 mm) and G2500 PWXL (7.8 × 300 mm) columns (Tosoh Corp.), connected in series, were eluted with 0.1 M NaNO_3_ at a flow rate of 0.6 mL/min, according to HP-SEC/TDA procedure. In the second case, TSK SW guardcolumn, TSK G3000 SWXL (7.5 × 300 mm) and TSK G2000 SWXL (7.5 × 300 mm) (Supelco), connected in series, were eluted with 0.2 M Na_2_SO_4_, at a flow of 0.5 mL/min, according to the EP indications. In both cases, processes were aided by Chromatography Manager Software Millennium–32 (Waters), with its GPC Empower option.

### 3.5. Sample Preparation

For HP-SEC/TDA analyses, samples were dissolved in a 0.1 M NaNO_3_ aqueous solution at about 10 mg/mL. 100 µL of each sample were injected. For HP-SEC all compounds were dissolved with the mobile phase, to a final concentration of 10 mg/mL and 20 µL were injected.

## Supplementary Materials

Supplementary materials can be accessed at: http://www.mdpi.com/1420-3049/20/03/5085/s1.
