# Supplementary material for: Determination of the Molecular Weight of Low-Molecular-Weight Heparins by Using High-Pressure Size Exclusion Chromatography on Line with a Triple Detector Array and Conventional Methods
Source: Molecules. 2015 Mar 19;20(3):5085–98. doi: 10.3390/molecules20035085 (PMC6272732; doi:10.3390/molecules20035085)
Supplement: Supplementary file 1 [file molecules-20-05085-s001.pdf]

Supplementary Material

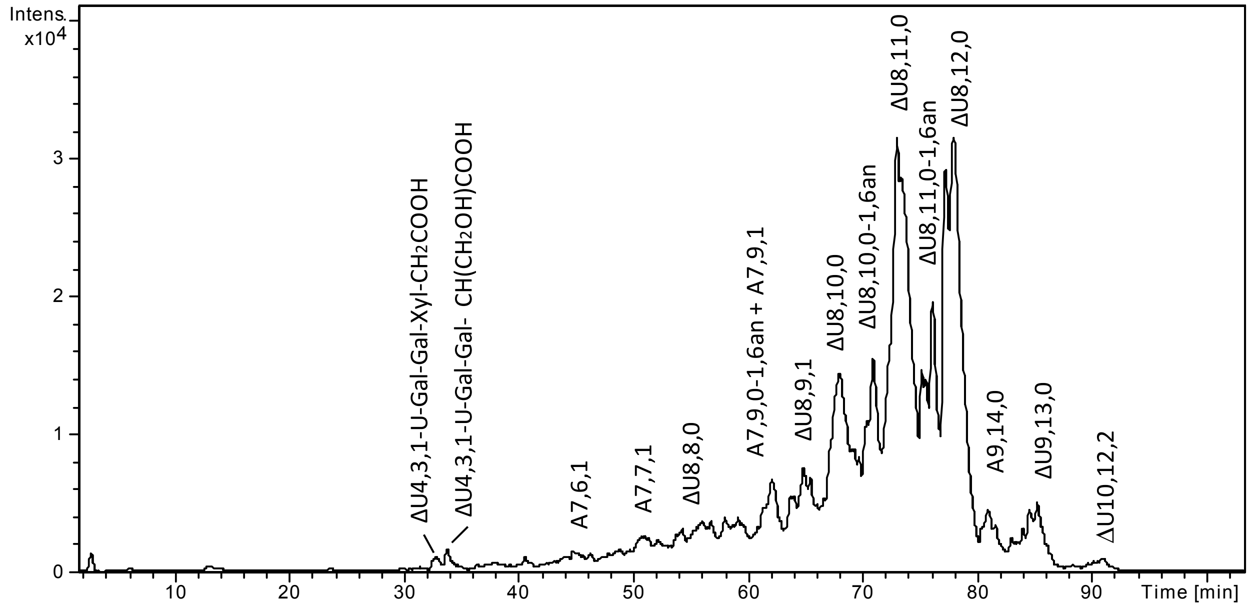

Figure S1. LC-MS profiles of enoxaparin octasaccharide fraction, isolated by Biogel P10 fractionation. Analysis was performed as previously reported [15]. LC conditions: column C18 100 × 2.1 mm, ODS 2.6 µm; eluents (A) 10 mM DBA + 10 mM CH_3COOH in H_2O and (B) 10 mM DBA + 10 mM CH_3COOH in CH_3OH; gradient elution 0 min 30%B, 10 min 35%B, 80 min 70%B, 120 min 90%B, 120 min 90%B, 125 min 30%B, 155 min 30%B; flow rate 0.1 mL/min. Structures are expressed by code consisting of three numbers (the numbers of monosaccharide residues, sulfate groups, and *N*-acetyl groups, respectively), preceded by the symbol ΔU to indicate that the first residue is 4,5-unsaturated uronic acid or by the symbol A for oligosaccharides starting with an aminosugar residue. These designations can be followed by 1,6an to indicate 1,6-anhydrohexosamine terminal residues.
